# Single‐cell RNA sequencing reveals inflammatory retinal microglia in experimental autoimmune uveitis

**DOI:** 10.1002/mco2.534

**Published:** 2024-04-07

**Authors:** Jiangyi Liu, Xingyun Liao, Na Li, Zongren Xu, Wang Yang, Hongxiu Zhou, Yusen Liu, Zhi Zhang, Guoqing Wang, Shengping Hou

**Affiliations:** ^1^ The First Affiliated Hospital of Chongqing Medical University Chongqing Key Laboratory for the Prevention and Treatment of Major Blinding Eye Diseases Chongqing Eye Institute Chongqing China; ^2^ Department of Medical Oncology Chongqing University Cancer Hospital Chongqing China; ^3^ Department of Laboratory Medicine Beijing Tongren Hospital Capital Medical University Beijing China; ^4^ Department of Kidney First Affiliated Hospital Third Military Medical University (Army Medical University) Chongqing China; ^5^ Beijing Institute of Ophthalmology Beijing Tongren Eye Center Beijing Tongren Hospital Capital Medical University Beijing Ophthalmology and Visual Sciences Key Laboratory Beijing China

**Keywords:** autoimmune uveitis, Ccl5, Cd74, microglia, single‐cell RNA sequencing

## Abstract

Autoimmune uveitis (AU) is a kind of immune‐mediated disease resulting in irreversible ocular damage and even permanent vision loss. However, the precise mechanism underlying dynamic immune changes contributing to disease initiation and progression of AU remains unclear. Here, we induced an experimental AU (EAU) model with IRBP_651‐670_ and found that day[D]14 was the inflammatory summit with remarking clinical and histopathological manifestations and the activation of retinal microglia exhibited a time‐dependent pattern in the EAU course. We conducted single‐cell RNA sequencing of retinal immune cells in EAU mice at four time points and found microglia constituting the largest proportion, especially on D14. A novel inflammatory subtype (*Cd74^high^ Ccl5^high^*) of retinal microglia was identified at the disease peak that was closely associated with modulating immune responses. In vitro experiments indicated that inflammatory stimuli induced proinflammatory microglia with the upregulation of CD74 and CCL5, and CD74 overexpression in microglia elicited their proinflammatory phenotype via nuclear factor‐kappa B signaling that could be attenuated by the treatment of neutralizing CCL5 antibody to a certain extent. In‐vivo blockade of Cd74 and Ccl5 effectively alleviated retinal microglial activation and disease phenotype of EAU. Therefore, we propose targeting CD74 and CCL5 of retinal microglia as promising strategies for AU treatment.

## Graphical abstract

Inflammatory retinal microglia with high expression of *Cd74* and *Ccl5* are identified at the disease peak of experimental autoimmune uveitis (EAU) by single‐cell RNA sequencing. Targeting Cd74 and Ccl5 effectively alleviated microglial activation and disease phenotype in the EAU model

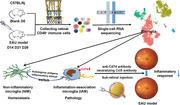

## 1 INTRODUCTION

Autoimmune uveitis (AU), one of the most sight‐threatening ocular diseases with complex etiologies, is characterized by severe intraocular inflammation. Currently, corticosteroids and immunosuppressants remain the mainstay of treatments for AU, however, they can cause a series of adverse effects and reduce patient compliance.^1^, ^2^ Comprehensive investigation on the pathogenesis of AU for developing novel therapies is urgently needed. Patients with AU often suffer from persistent inflammation caused by autoimmune‐mediated damage to the targeted retinal and uveal tissues.^3^, ^4^ Utilizing multi‐omics approaches, accumulated studies have demonstrated immune disturbance in the eyes of AU cases.^5^, ^6^, ^7^ However, the precise mechanism underlying dynamic alterations of the intraocular immune microenvironment in the initiation and progression of AU is still not fully elucidated.

The application of single‐cell RNA sequencing (scRNA‐seq) technology to divide complex tissues into finer cell subsets at the transcriptome level helps us to identify disease‐related cell subgroups under pathological conditions to develop new therapies.^8^, ^9^, ^10^ scRNA‐seq has been used to obtain subtle information on the local immune microenvironment for multiple ocular diseases, including diabetic retinopathy and age‐related macular degeneration.^11^, ^12^ Currently, extensive studies have explored peripheral immune features for AU and disease‐related animal models that have provided potential strategies for its pathogeny, however, the details of ocular immune characteristics for AU still remain lacking.^13^, ^14^

Microglia as resident immune cells in the eye, play a pivotal role in the homeostatic maintenance of retinal immunoregulation.^15^, ^16^ Under the pathologies of multiple diseases, microglia with distinct features and functions are found to be involved in various pathophysiological processes.^17^, ^18^, ^19^ Many studies have revealed the essential roles of retinal microglia in the AU process, including antigen presentation and amplifying inflammatory responses during the disease process.^20^, ^21^ As a critical regulator of immune responses, retinal microglia can be a potential therapeutic target for AU. However, microglial subsets under AU pathology have not been well characterized and their functions still require further study.

Here, we used an experimental AU (EAU) mouse model developed with interphotoreceptor retinoid‐binding protein 651–670 (IRBP_651–670_) to explore the pathogenesis of AU, and determined day[D]14 as the inflammatory peak of EAU characterized by obvious clinical and pathological changes with remarking microglial activation. Through the construction of single‐cell transcriptional profiles of retinal immune cells at different time points in the EAU course, we identified an inflammation‐associated microglial subpopulation with high expression of *Cd74* and *Ccl5* on D14, which was actively involved in the pathways related to modulating immune and inflammatory responses. In vitro experiments confirmed the enrichment of CD74 and CCL5 in microglia after inflammatory stimulation and further results indicated that CD74 overexpression activated microglia into proinflammatory phenotype via the nuclear factor‐kappa B (NF‐κB) pathway, which could be reversed by the blockade of CCL5. Moreover, subretinal administration of neutralizing antibodies against Cd74 and Ccl5 effectively alleviated microglial activation and disease phenotype of EAU. Thus, we suggested that targeting CD74 and CCL5 of retinal microglia might be novel targets for AU treatment.

## 2 RESULTS

### 2.1 Dynamic characteristics of EAU phenotype

**FIGURE 1:**
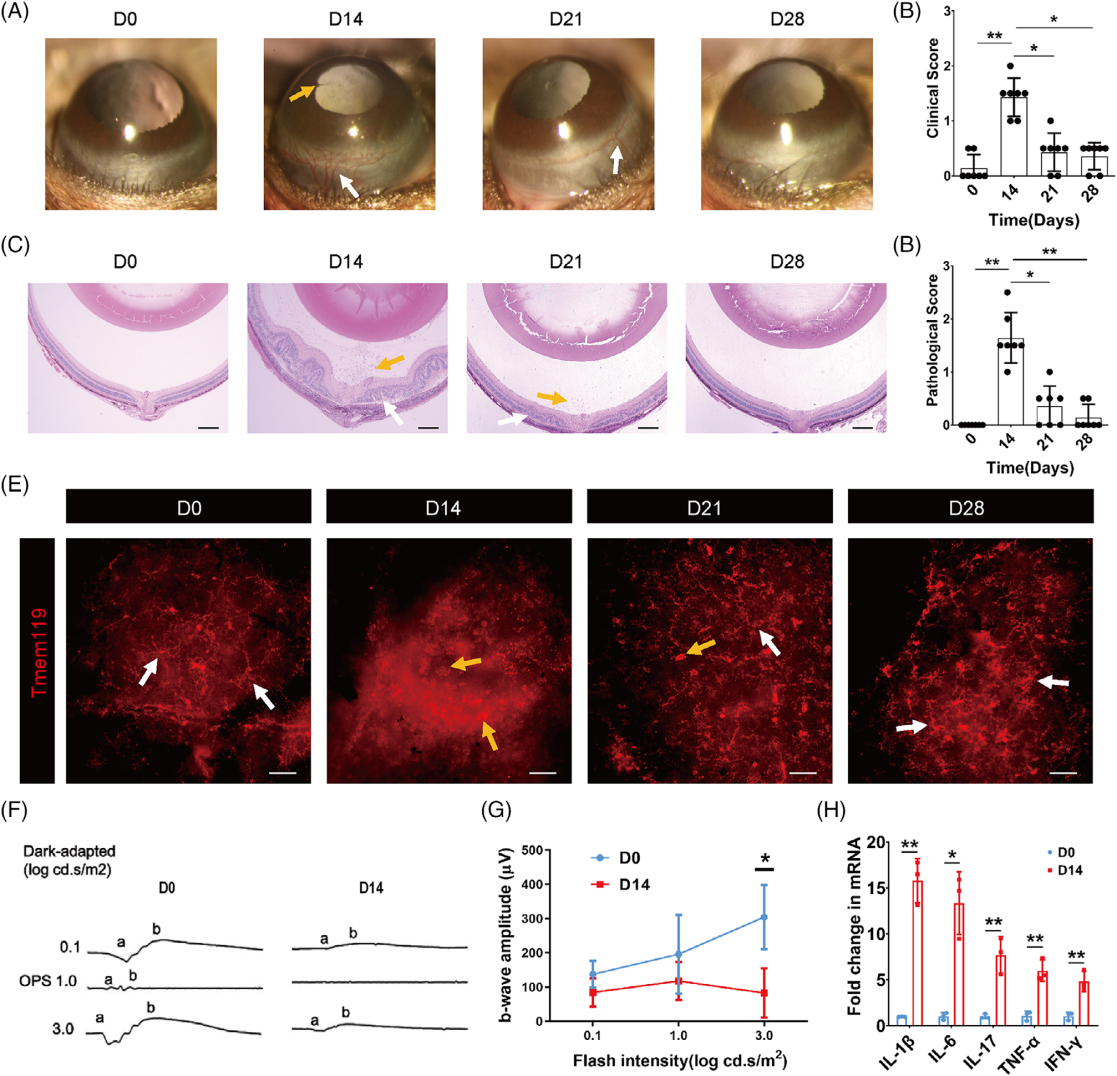
Identification of disease phenotype at different time points during experimental autoimmune uveitis (EAU). (A, B) The clinical symptoms and scores during the EAU process. *n* = 7 mice per group. (white arrows, conjunctival and/or ciliary congestion; yellow arrows, synechiae). (C, D) Retinal histopathological staining and scores during the EAU process. *n* = 7 mice per group. (scale bar, 200 µm; yellow arrows, infiltration of inflammatory cells; white arrows, retinal folds). (E) Retinal microglia (Tmem119^+^) staining during the EAU process. (scale bar, 20 µm; yellow arrows, amoeboid microglia; white arrows, ramified microglia). (F, G) Retinal function measurements in the D0 group and D14 EAU group. (H) Retinal mRNA expression of proinflammatory cytokines in the D0 group and D14 EAU group. * *p* < 0.05, ** *p* < 0.01, NS: no significance.

To investigate the dynamic alterations and immune responses in EAU mice injected with IRBP_651‐670_, we first assessed the clinical manifestations and pathological changes at different time points (D7, D14, D21, and D28). The mice on D0 without IRBP_651‐670_ immunization were defined as the healthy control. The slit‐lamp results showed the most remarkable inflammatory signs of EAU mice on D14, including conjunctival hyperemia and posterior synechiae, which gradually recovered over the subsequent time points (Figure 1A,B). The histopathological staining also revealed extensive immune cell infiltration and obvious plication in the EAU retina on D14, which were ameliorated on D21 and D28 (Figure 1C,D). No significant difference was observed between D0 and D7 (Figure S1A–D). Next, we performed immunofluorescence staining to detect retinal microglial activation which is regarded as a key feature of EAU initiation.^21^ The results demonstrated that relatively static microglia (Tmem119^+^) with a highly ramified morphology on D0 and D7 transformed into an exclusively activated amoeboid morphology on D14, and then restored ramified again on D21 and D28 as the disease progressed, suggesting that microglial activation exhibited a time‐dependent pattern during EAU (Figure 1E and Figure S1E). Together, these results indicated D14 as the summit of inflammatory responses and disease severity during EAU progression.

Furthermore, we assessed the retinal function of EAU mice on D0 and D14 by electroretinogram (ERG) and found that the dark‐adapted amplitudes of the a‐ and b‐wave decreased on D14 which could not be elicited (Figure 1F). The amplitude of the b‐wave was significantly reduced on D14 compared to D0, suggesting that robust inflammatory responses induced severe retinal damage in EAU mice (Figure 1G). Real‐time quantitative PCR (RT‐qPCR) results also revealed that the mRNA level of a series of proinflammatory cytokines was significantly upregulated in the EAU retina on D14 (Figure 1H). Therefore, these data indicated that inflammatory responses of EAU peaked on D14 after IRBP_651‐670_ immunization.

### 2.2 Construction of single‐cell retinal immune atlas in EAU process

**FIGURE 2:**
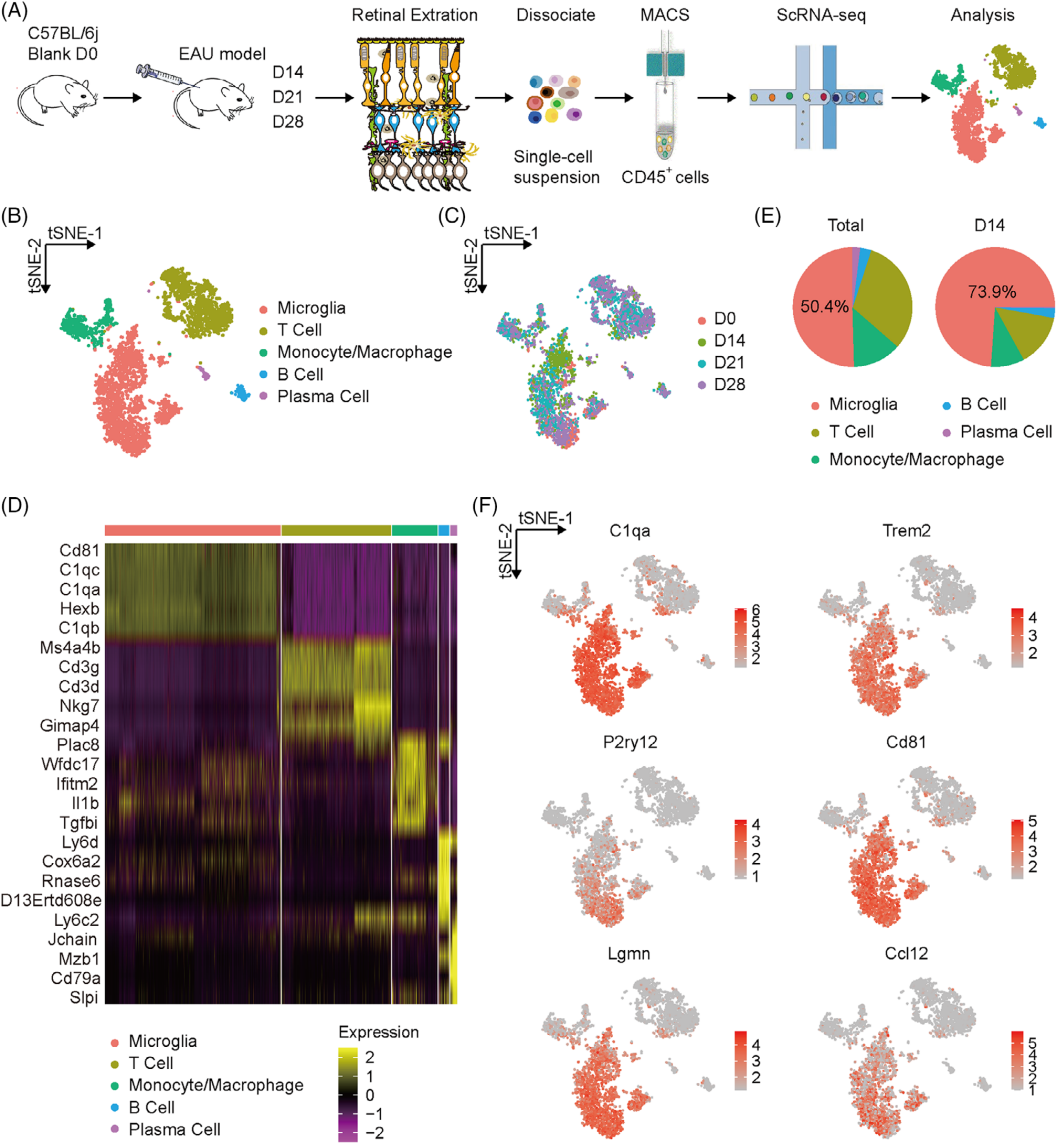
Single‐cell RNA sequencing (scRNA‐seq) atlas of retinal immune cells during experimental autoimmune uveitis (EAU). (A) The schematic diagram of scRNA‐seq design. (B) t‐distributed stochastic neighbor embedding (t‐SNE) plot of retinal immune cells during EAU colored by cell type. (C) A t‐SNE plot of retinal single cells during EAU colored by time points. (D) Heatmap of the top five differentially expressed genes (DEGs) expressed in each immune cell type during EAU. (E) Pie plots of the percentage of each immune cell type among total cells and cells on D14, respectively. (F) t‐SNE plots of several genes expressed in microglial clusters during EAU.

To characterize the immune landscape involved in the EAU retina, we sorted all CD45^+^ immune cells of retinal tissues extracted from EAU mice during disease progression for single‐cell suspensions and performed massively parallel scRNA‐seq using the 10×Genomics platform (Figure 2A and Figure S2A). Here, four time points in the EAU process were assessed: D0 (healthy control), D14 (at the peak of inflammatory responses), D21, and D28 (at the late stage of inflammatory responses). Finally, a total of 3,561 cells were retained and enrolled in the following analysis. These cells were subjected to unsupervised graph‐based clustering and identified according to classical lineage markers, including microglia expressing *P2ry12* and *Tmem119*, T cell expressing *Cd3d* and *Cd3g*, monocyte/macrophage expressing *Ccr2* and *Ms4a4c*, B cell expressing *Ly6d* and *Mzb1*, and plasma cell expressing *Sdc1* (Figure 2B,C, and Figure S2B; Table S1). Different cell types exhibited unique expression patterns under EAU pathology (Figure 2D; Table S2).

Next, we analyzed the proportion of each cell type over time and found that microglia composed the largest population and were highly enriched on D14, suggesting that they were closely associated with inflammatory responses and disease severity of EAU (Figure 2E). Except canonical microglial marker genes representing homeostasis or activation (such as *P2ry12*, *C1qa*, and *Trem2*), this microglial cluster also expressed several specific genes, including *Ccl12* involved in leukocyte chemotaxis, *Cd81*, and *Lgmn* concerning leukocyte migration (Figure 2F). Furthermore, the number of differentially expressed genes (DEGs) reached the maximum on D14 compared to D0 in the microglial cluster, suggesting remarking changes in the immune phenotype of retinal microglia at the EAU inflammatory peak (Figure S2C).

### 2.3 Identification of disease‐associated microglial subtype at EAU inflammatory peak

**FIGURE 3:**
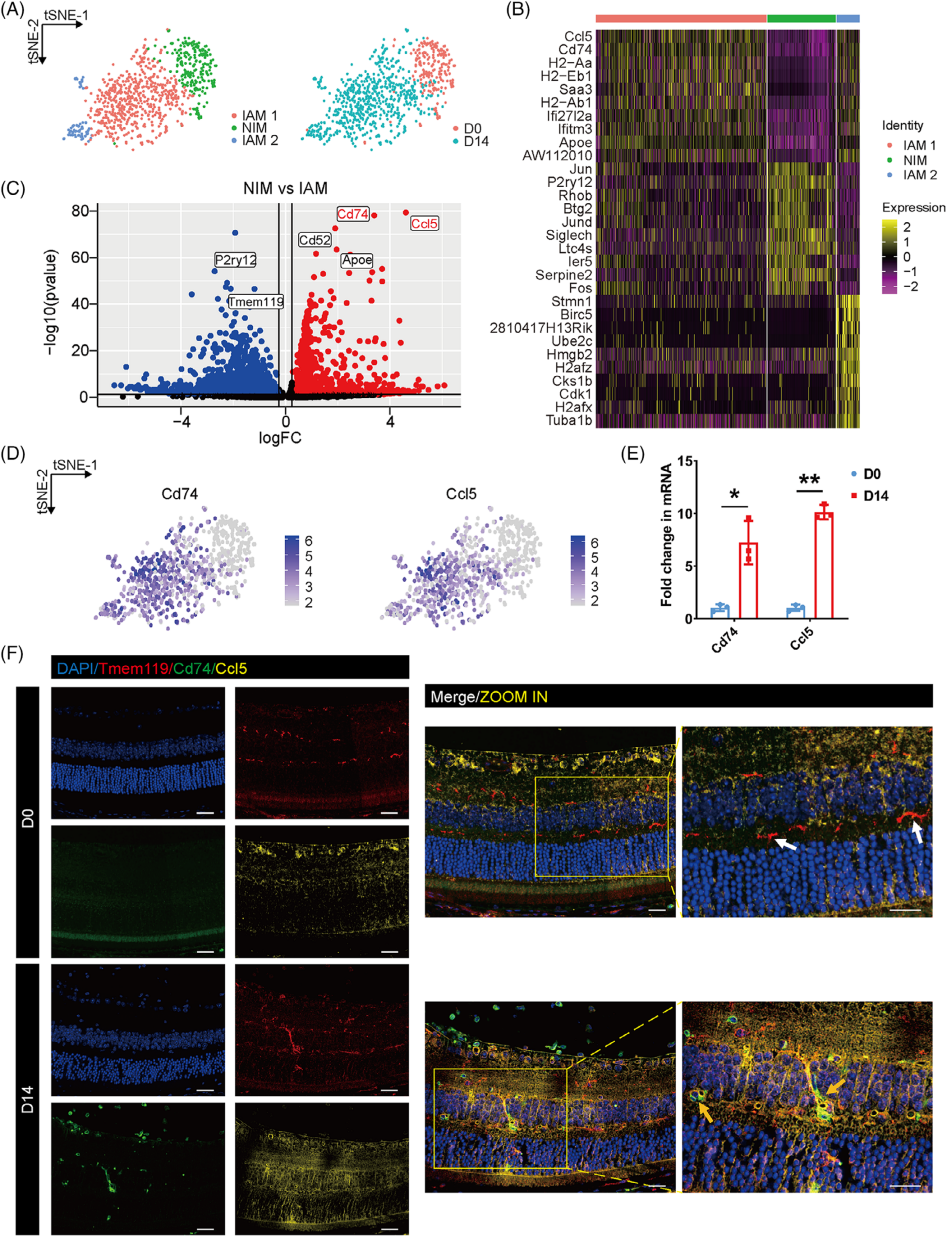
Identification of disease‐associated retinal microglia at the experimental autoimmune uveitis (EAU) peak. (A) t‐distributed stochastic neighbor embedding (t‐SNE) plots of microglial cells on D0 and D14 split by subpopulations and time points. (B) Heatmap of the top 10 differentially expressed genes (DEGs) expressed in each microglial subpopulation on D0 and D14. (C) Volcano plot of the DEGs between non‐inflammatory microglia (NIM) and inflammation‐associated microglia (IAM). (D) t‐SNE plots of *Cd74* and *Ccl5* expressed in microglial subpopulations on D0 and D14. (E) Retinal mRNA expression of *Cd74* and *Ccl5* in the D0 group and D14 EAU group. (F) Representative images of retinal microglia (Tmem119^+^) co‐stained with Cd74 and Ccl5 in the D0 group and D14 EAU group. (scale bar, 20 µm; white arrows, Cd74^−^ Ccl5^−^ microglia; yellow arrows, Cd74^+^ Ccl5^+^ microglia). * *p* < 0.05, ** *p* < 0.01.

To explore the role of retinal microglia at the EAU inflammatory peak, we extracted microglial cells on D0 and D14 for further study. After unsupervised graph‐based clustering, these cells were divided into three subpopulations with respective expression patterns (Figure 3A,B and Table S3). Subcluster 2 was defined as the non‐inflammatory microglia (NIM) due to its existence primarily on D0 with high expression of homeostatic genes including *P2ry12* and *Tmem119* (Figure S3A). Whereas the vast majority of microglial cells on D14 specifically expressing activation genes (*Apoe* and *Cd52*) were identified as inflammation‐associated microglia (IAM) and comprised subcluster 1 and 3 (IAM1 and IAM2). We further found that these two IAM subgroups shared analogous gene expression patterns with correlation coefficients greater than 0.5, indicating their similarities in signatures and functions (Figure S3B).

Thus, we first merged IAM1 and IAM2 as one population to conduct DEG analysis compared to the NIM cluster (Figure 3C). Genes concerning receptor‐mediated endocytosis (*Siglech*) and circadian rhythm (*Zfhx3*) are specifically expressed in NIM, whereas genes involved in necrotic cell death (*Fth1*) and cytoplasmic translation (*Rpl32*) are highly expressed in IAM, suggesting the activation state of IAM (Figure S3C). Notably, this IAM population shared a specific high expression of *Cd74* and *Ccl5*, the top two upregulated DEGs in IAM, suggesting that these two markers might be closely correlated with microglial activation on D14 (Figure 3D). RT‐qPCR results confirmed a significant increase of *Cd74* and *Ccl5* mRNA in the EAU retina on D14 (Figure 3E). Importantly, we observed the specific presence of these Cd74^+^ Ccl5^+^ microglia (Tmem119^+^) in the EAU retina on D14 through immunofluorescence staining (Figure 3F and Figure S3D). We also found brain microglia with high expression of *Cd74* and *Ccl5* in single‐cell data of experimental autoimmune encephalomyelitis (EAE) mice, a mouse model of multiple sclerosis (MS), suggesting that the upregulation of CD74 and CCL5 in microglia might be supposed to contribute to the pathogenesis of a series of autoimmune diseases including AU and MS (Figure S3E).^22^

These results indicated the specific presence of *Cd74^high^ Ccl5^high^* microglial subsets highly correlated with inflammatory responses at the disease peak of EAU.

### 2.4 Functional analysis of inflammation‐associated microglia at EAU inflammatory peak

**FIGURE 4:**
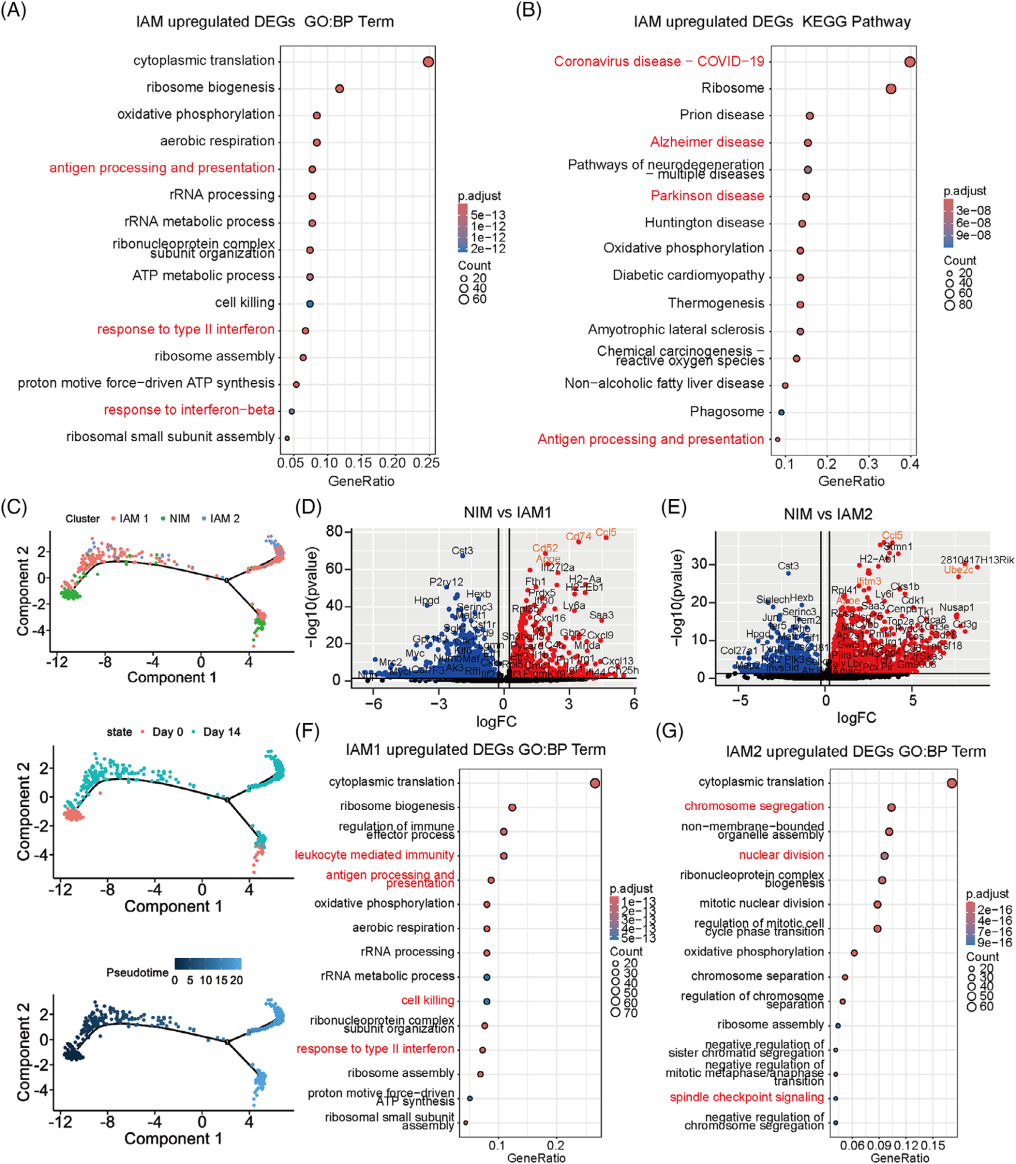
Functional analysis of retinal microglia subpopulations in experimental autoimmune uveitis (EAU). (A, B) Dot plots of the top (A) Gene Ontology (GO) BP terms and (B) Kyoto Encyclopedia of Genes and Genomes (KEGG) pathways were calculated by using upregulated differentially expressed genes (DEGs) in inflammation‐associated microglia (IAM) compared with non‐inflammatory microglia (NIM). (C) Plots of the pseudo‐time trajectory of microglia subpopulations on D0 and D14. (D) Volcano plot of the DEGs between IAM1 and NIM. (E) Volcano plot of the DEGs between IAM2 and NIM. (F, G) Dot plots of the top GO: BP terms calculated by using upregulated DEGs in (F) IAM1 and (G) IAM2 subpopulations compared with NIM. BP, biological process.

Next, we focused on the function of the *Cd74* ^high^ *Ccl5* ^high^ IAM population. DEGs upregulated in IAM were highly enriched in the ‘antigen processing and presentation’ and ‘response to type II interferon’ pathways by Gene Ontology (GO) analysis, and were actively implicated in the pathways related to several immune‐related diseases (such as coronavirus disease 2019, Alzheimer's disease, and Parkinson's disease) by Kyoto Encyclopedia of Genes and Genomes (KEGG) analysis (Figure 4A,B). These results indicated that this specific IAM population with high expression of *Cd74* and *Ccl5* could be a critical mediator of immune responses at the peak of EAU inflammation.

To further comprehend the differential features of IAM subpopulations in the EAU process, we first conducted a pseudo‐time analysis and found that retinal microglia underwent a transitional state (IAM1) from NIM to IAM2 (Figure 4C). We next compared the DEGs of IAM1 and IAM2 with NIM, respectively (Figure 4D,E). The data showed that *Ccl5* and *Apoe* were highly expressed in both IAM1 and IAM2 groups compared with NIM. *Cd74* and *Cd52* were specifically expressed in IAM1, whereas *Ifitm3* and *Ube2c* were enriched in IAM2. A subsequent GO analysis demonstrated that DEGs upregulated in IAM1 were enriched in the ‘response to type II interferon’, ‘cell killing’, and ‘leukocyte mediated immunity’ pathways, whereas the role of IAM2 was actively implicated in the ‘nuclear division’ and ‘spindle checkpoint signaling’ pathways (Figure 4F,G). These data suggested that the IAM1 subtype exhibited a proinflammatory phenotype implicated in pathways triggering inflammatory responses, and IAM2 primarily contributed to cell proliferation and immunoregulation. Moreover, KEGG analysis suggested the upregulation of the ‘antigen processing and presentation’ and immune disease‐associated pathways in both IAM1 and IAM2 compared to NIM (Figure S4A,B).

In summary, these results indicated the adequate involvement of IAM subpopulations in inflammatory immunity at the disease peak of EAU.

### 2.5 Proinflammatory phenotype of microglia elicited by Cd74/Ccl5/NF‐κB signaling in vitro

**FIGURE 5:**
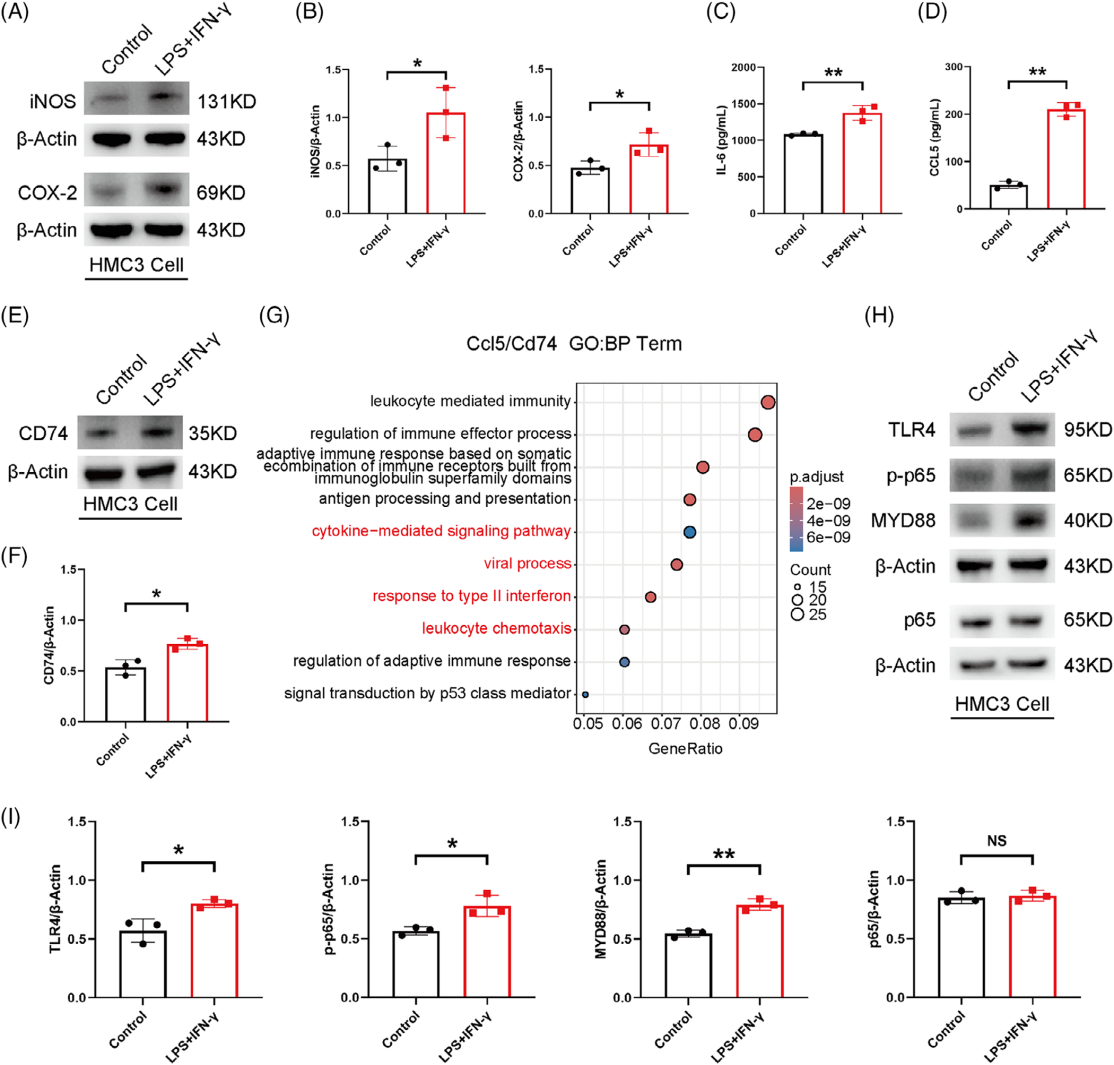
Microglial alterations under inflammation stimulation in vitro. (A, B) The protein expression of proinflammatory microglial markers (cyclooxygenase‐2 [COX‐2] and inducible nitric oxide synthase [iNOS]) in the Control and lipopolysaccharide (LPS) + interferon‐gamma (IFN‐γ) human microglial clone 3 (HMC3) cells. (C) The protein expression of interleukin 6 (IL‐6) in the Control and LPS + IFN‐γ HMC3 cells measured by enzyme‐linked immunosorbent assay (ELISA). (D) The protein expression of CCL5 in the Control and LPS + IFN‐γ HMC3 cells measured by ELISA. (E, F) The protein expression of CD74 in the Control and LPS + IFN‐γ HMC3 cells. (G) Dot plot of the Gene Ontology (GO): BP terms (containing *Cd74* and/or *Ccl5*) calculated by using upregulated differentially expressed genes (DEGs) in inflammation‐associated microglia (IAM) compared with non‐inflammatory microglia (NIM). (H, I) The protein expression of toll‐like receptor 4 (TLR4) signaling factors in the Control and LPS + IFN‐γ HMC3 cells. * *p* < 0.05, ** *p* < 0.01, NS: no significance.

To probe the role of *Cd74^high^ Ccl5^high^* IAM in EAU, we utilized human microglial clone 3 (HMC3) cells and murine BV2 cells under inflammatory stimulation in vitro to simulate microglial activation at EAU inflammatory peak. After the stimulation of lipopolysaccharide (LPS) (1 µg) and interferon‐gamma (IFN‐γ) (0.5 µg) for 24 h, the protein level of inflammatory factors (including inducible nitric oxide synthase [iNOS], cyclooxygenase‐2 [COX‐2], and interleukin 6 [IL‐6]) was significantly increased in HMC3 cells, indicating the proinflammatory phenotype of microglia (Figure 5A–C). The expression of CD74 and CCL5 in inflammatory HMC3 cells was also markedly upregulated, similar to the enrichment of *Cd74* and *Ccl5* in the IAM cluster on D14 (Figure 5D–F). Given that both *Cd74* and *Ccl5* were implicated in the ‘cytokine‐mediated signaling pathway’ and ‘response to type II interferon’ pathway enriched in IAM, we focused on the toll‐like receptor 4 (TLR4) signaling pathway, which is reported to be rapidly activated in microglia primed by IFN‐γ with the stimulation of TLR4 ligands and results in excessive release of proinflammatory cytokines (Figure 5G).^23^ We detected the expression of classical factors in this pathway, including TLR4, MYD88, NF‐κB p65, and NF‐κB p‐p65. The protein level of these key factors was significantly elevated in HMC3 cells after LPS and IFN‐γ stimulation for 24 h (Figure 5H,I). Furthermore, we observed consistent results in BV2 cells after LPS (1 µg) stimulation for 24 h (Figure S5A–F). These results suggested that proinflammatory microglia possessed high expression of CD74 and CCL5, and were actively involved in the activation of the TLR4 signaling pathway.

**FIGURE 6:**
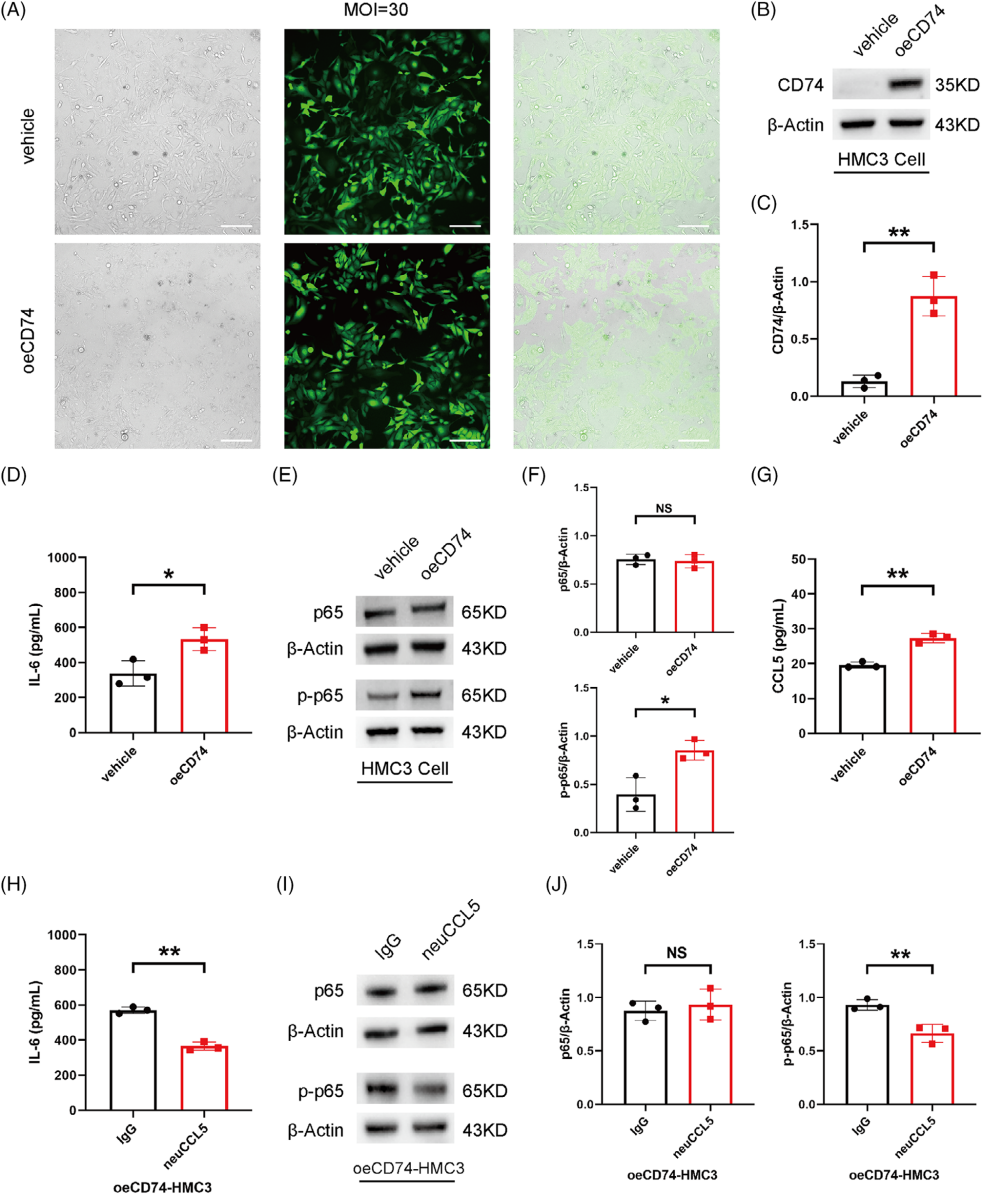
Molecular mechanism of CD74 and CCL5 in microglia phenotype. (A) Human microglial clone 3 (HMC3) cells were exposed to overexpressing CD74 (oeCD74) and vehicle lentivirus, and transfection efficiency was assessed by fluorescence microscope. (scale bar, 100 µm). (B, C) The protein expression of CD74 in the vehicle and oeCD74 HMC3 cells. (D) The protein expression of interleukin 6 (IL‐6) in the vehicle and oeCD74 HMC3 cells measured by enzyme‐linked immunosorbent assay (ELISA). (E, F) The protein expression of nuclear factor‐kappa B (NF‐κB) factors in the vehicle and oeCD74 HMC3 cells. (G) The protein expression of CCL5 in the vehicle and oeCD74 HMC3 cells measured by ELISA. (H) The protein expression of IL‐6 in the immunoglobulin G (IgG) and neuCCL5 oeCD74‐HMC3 cells measured by ELISA. (I, J) The protein expression of NF‐κB factors in the IgG and neuCCL5 oeCD74‐HMC3 cells. * *p* < 0.05, ** *p* < 0.01, NS: no significance.

CD74 overexpression in macrophages can modulate NF‐κB activation to promote the inflammatory response, therefore, we transfected HMC3 cells with overexpressing CD74 (oeCD74) and vehicle lentivirus to determine whether CD74 in microglia could share the equivalent effect.^24^ We successfully overexpressed CD74 in HMC3 cells at first (Figure 6A–C). The expression of NF‐κB p‐p65 and IL‐6 was significantly upregulated in the oeCD74 group, suggesting that CD74 could enhance the induction of proinflammatory microglia through the activation of the NF‐κB signaling (Figure 6D–F). Notably, we observed the upregulation of CCL5 secretion in the oeCD74‐HMC3 cells, suggesting that CCL5 could be a downstream molecule in microglia affected by CD74, similar to a previous study found in astrocytes (Figure 6G).^25^ We further assessed whether CCL5 was involved in the CD74‐mediated proinflammatory phenotype of microglia by utilizing neutralizing CCL5 (neuCCL5) antibody. The data showed that the treatment of neuCCL5 antibody (1 µg) reduced the protein level of NF‐κB p‐p65 and IL‐6, suggesting that the blockade of CCL5 reversed oeCD74‐elicited microglial polarization shifting to proinflammatory phenotype to a certain extent (Figure 6H–J). Together, these results indicated that the proinflammatory phenotype of microglia might be regulated by Cd74/Ccl5/NF‐κB signaling during EAU progression.

In summary, these results suggested that *Cd74^high^ Ccl5^high^* IAM might be responsible for robust inflammation in an activated NF‐κB signaling manner at EAU disease peak.

### 2.6 Potential therapeutic effects of inhibiting *Cd74* and *Ccl5* in vivo

**FIGURE 7:**
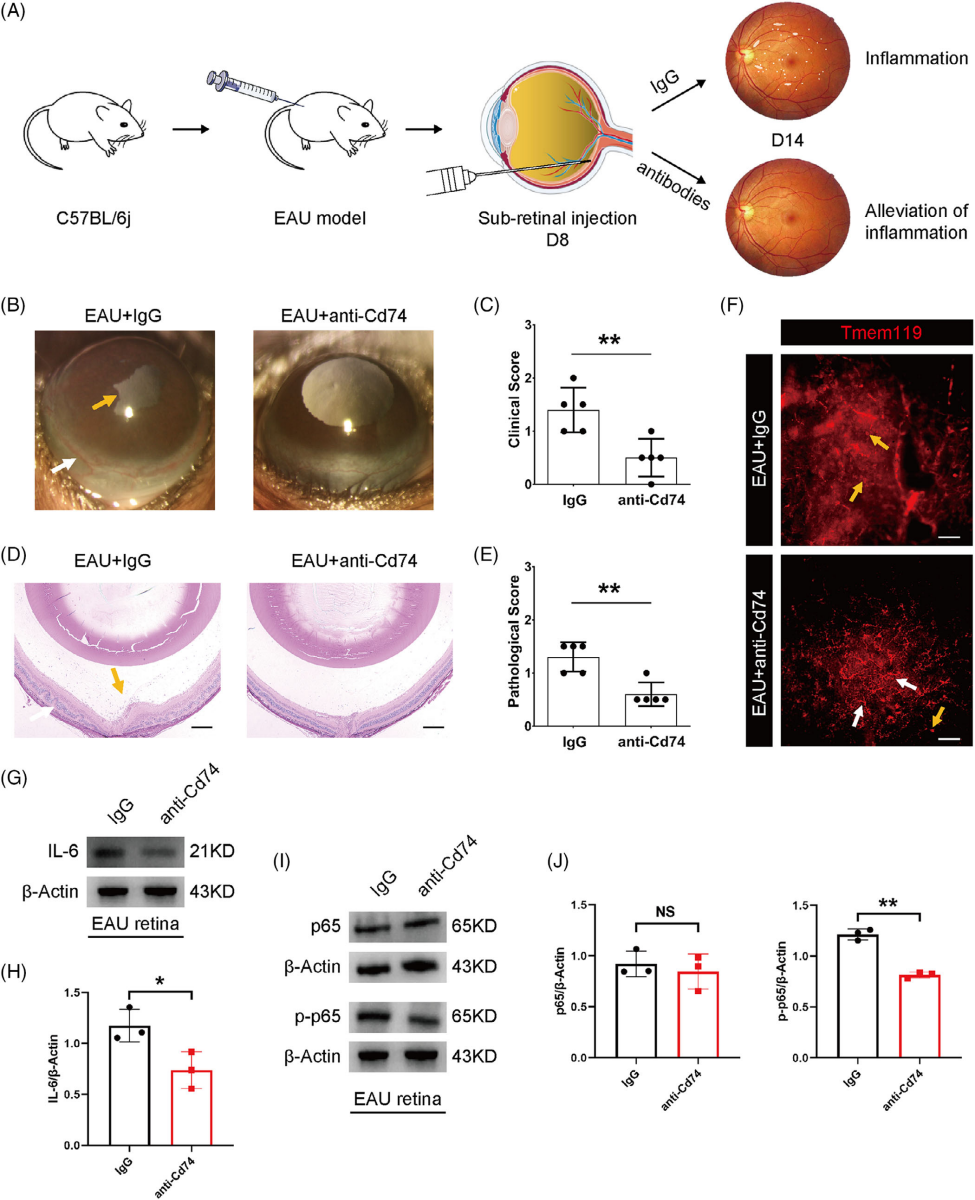
Inhibiting Cd74 ameliorated experimental autoimmune uveitis (EAU) disease phenotype. (A) Schematic diagram of subretinal injection of the polyclonal anti‐Cd74 antibody or neutralizing Ccl5 (neuCcl5) antibody. (B, C) The clinical symptoms and scores in the EAU + immunoglobulin G (IgG) and EAU + anti‐Cd74 groups on D14. *n* = 5 mice per group. (white arrows, conjunctival and/or ciliary congestion; yellow arrows, synechiae). (D, E) Retinal histopathological staining and scores in the EAU + IgG and EAU + anti‐Cd74 groups on D14. *n* = 5 mice per group. (scale bar, 200 µm; yellow arrows, infiltration of inflammatory cells; white arrows, retinal folds). (F) Retinal microglia (Tmem119^+^) staining in the EAU + IgG and EAU + anti‐Cd74 groups on D14. (scale bar, 20 µm; yellow arrows, amoeboid microglia; white arrows, ramified microglia). (G, H) The retinal protein expression of interleukin 6 (IL‐6) in the EAU + IgG and EAU + anti‐Cd74 groups. (I, J) The retinal protein expression of nuclear factor‐kappa B (NF‐κB) factors in the EAU + IgG and EAU + anti‐Cd74 groups. * *p* < 0.05, ** *p* < 0.01, NS: no significance.

To explore whether targeting IAM characteristic molecules (*Cd74* and *Ccl5*) can alleviate disease phenotype at the peak of EAU inflammation, we delivered a polyclonal anti‐Cd74 antibody or neutralizing Ccl5 (neuCcl5) antibody into EAU mice on D8 after IRBP_651‐670_ immunization through subretinal injection (Figure 7A). The anti‐Cd74 antibody‐treated EAU mice exhibited lighter conjunctival hyperemia and synechiae, and less retinal plication and infiltration of inflammatory cells than the immunoglobulin G (IgG)‐treated EAU mice (Figure 7B–E). We also observed a decline of amoeboid retinal microglia (Tmem119^+^) after the treatment of anti‐Cd74 antibody, suggesting that microglial activation was effectively suppressed by the blockade of Cd74 (Figure 7F). The expression of IL‐6 and p‐p65 was significantly reduced in the EAU mice treated with anti‐Cd74 antibody (Figure 7G–J). These results showed that targeting Cd74 could markedly reduce microglial activation and weaken EAU disease phenotype through diminishing NF‐κB signaling.

**FIGURE 8:**
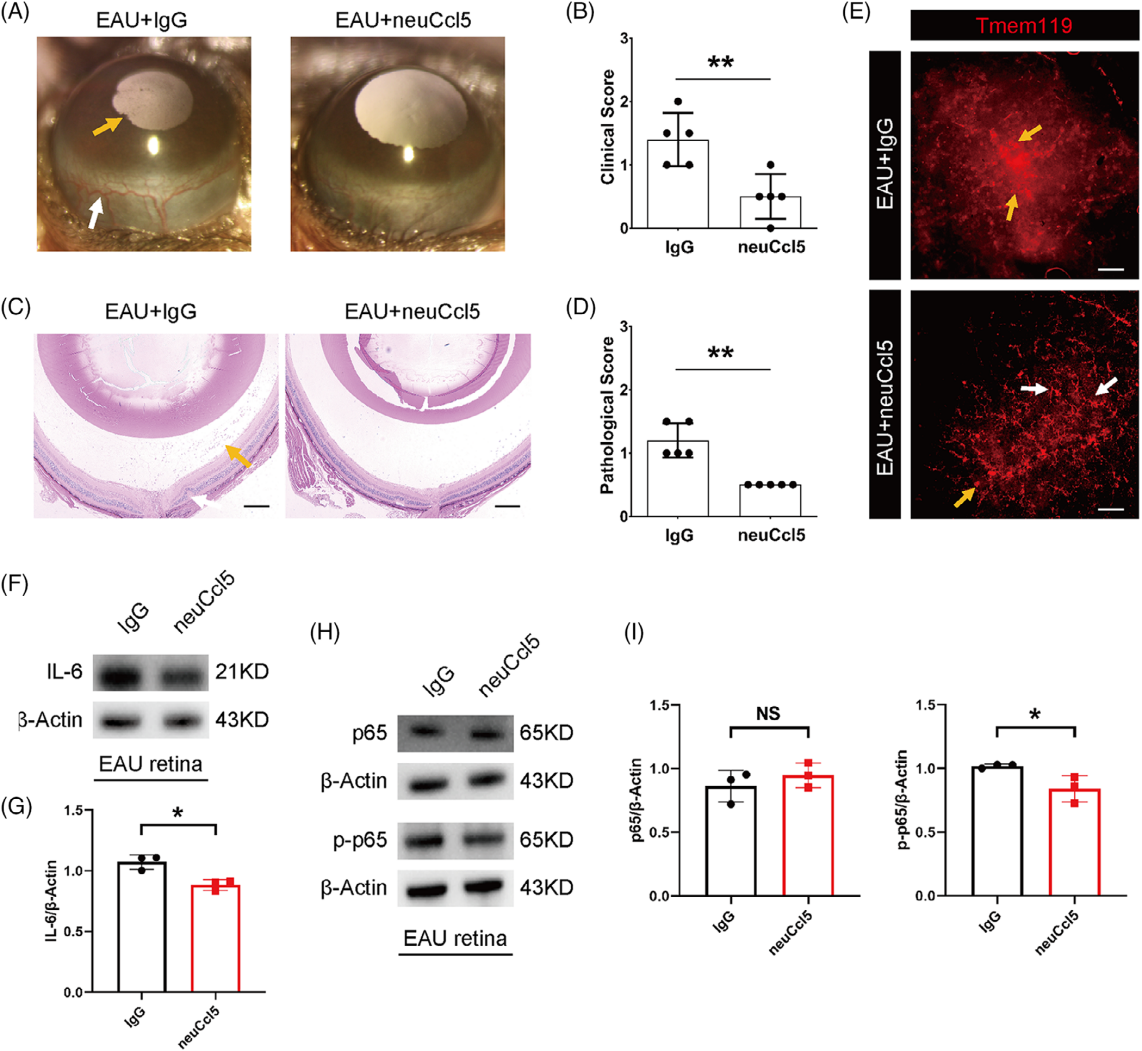
Potential therapeutic role of suppressing Ccl5 in experimental autoimmune uveitis (EAU). (A, B) The clinical symptoms and scores in the EAU + immunoglobulin G (IgG) and EAU + neutralizing Ccl5 (neuCcl5) groups on D14. *n* = 5 mice per group. (white arrows, conjunctival and/or ciliary congestion; yellow arrows, synechiae). (C, D) Retinal histopathological staining and scores in the EAU + IgG and EAU + neuCcl5 groups on D14. *n* = 5 mice per group. (scale bar, 200 µm; yellow arrows, infiltration of inflammatory cells; white arrows, retinal folds). (E) Retinal microglia (Tmem119^+^) staining in the EAU + IgG and EAU + neuCcl5 groups on D14. (scale bar, 20 µm; yellow arrows, amoeboid microglia; white arrows, ramified microglia). (F, G) The retinal protein expression of interleukin 6 (IL‐6) in the EAU + IgG and EAU + neuCcl5 groups. (H, I) The retinal protein expression of nuclear factor‐kappa B (NF‐κB) factors in the EAU + IgG and EAU + neuCcl5 groups. * *p* < 0.05, ** *p* < 0.01, NS: no significance.

Similarly, the neuCcl5 antibody‐treated group also exhibited attenuated hyperemia reduced synechia of the anterior segment, and milder pathological changes in the retina on D14 (Figure 8A–D). As expected, the activation of microglia and NF‐κB factors was reversed in the retina of EAU mice with the application of neuCcl5 antibody (Figure 8E–I). Together, these data indicated that the blockade of Ccl5 could relieve microglial activation and the inflammation of the EAU model.

In conclusion, these results suggested that these two specific molecules of IAM could be potent therapeutic targets for AU.

## 3 DISCUSSION

AU refers to a complex collection of heterogeneous inflammatory diseases as one of the main blindness causes that can cause a set of serious complications and sequelae. Current mainstay therapies for AU bring unsatisfactory clinical benefits and are accompanied by a series of side effects leading to poor patient compliance.^26^, ^27^ Thus, the pathogenesis of AU needs further investigation to provide new potential targets. Extensive research has demonstrated full‐blown disease developing by D14‐21 after IRBP immunization utilizing the EAU model that is widely used for human endogenous posterior uveitis.^28^, ^29^ In this study, we determined D14 as the robust inflammatory peak during disease progression of EAU mice developed with IRBP_651‐670_, manifested as remarking clinical and pathological signs, accumulated generation of proinflammatory cytokines, as well as excessive microglial activation.

Microglia are regarded as immune watchdogs to survey the immune microenvironment in normal and diseased retinas and have been reported as a vital initiator in the disease onset of EAU.^21^ Interestingly, we found that the activation of retinal microglia exhibited a temporal correlation with the inflammation level of EAU: a substantial increase of activated microglia with amoeboid morphology at the peak of disease (D14), with a subsequent decrease upon disease gradual resolution. Our scRNA‐seq data of EAU retinal immune cells also indicated that microglia were the most numerous immune cell types observed during EAU, especially at the inflammatory peak. Therefore, microglia play a pivotal role not only in the initiation of EAU but also in the following inflammation progress, suggesting they can be an essential therapeutic target for AU.

Under AU pathology, microglia are primed and activated by autologous antigens and inflammatory stimuli, which promote the disruption of the blood‐retinal barriers and provoke a wide range of immunocyte infiltration into the retina, resulting in severe local inflammation.^30^, ^31^ To explore the precise mechanism underlying microglial activation during EAU, we scrutinized microglial cluster and identified a new subtype of microglia with high expression of *Cd74* and *Ccl5*. This subgroup displayed a proinflammatory phenotype with enrichment of activated markers and primarily occupied the EAU retina at the disease peak, which was actively involved in the pathways associated with the inflammatory response and immunoregulation, indicating that they might be responsible for the robust inflammation during EAU. We also found high expression of *Cd74* and *Ccl5* in brain microglia of EAE mice, indicating that these two characteristic molecules expressed in microglia might contribute to the occurrence and progression of multiple autoimmune diseases with similar pathology.^22^ However, the presence and function of this special microglial subtype still need further investigation using more scRNA‐seq datasets of other diseases.

We noticed *Cd74* and *Ccl5* actively implicated in the pathways related to inflammatory chemotaxis and interferon‐mediated responses, and focused on TLR signaling, especially TLR4‐stimulated transcription factors (NF‐κB), which has been reported to exaggerate immune responses of microglia and play a crucial role in the ‘super‐activation’ of microglia priming by IFN‐γ.^23^, ^32^, ^33^ In vitro experiments found that remarkable activation of NF‐κB signaling and enrichment of CD74 and CCL5 in microglia responding to inflammatory stimuli. CD74 activated microglia into proinflammatory phenotype via upregulating the phosphorylation of NF‐κB protein p65 and expression of inflammatory cytokine, suggesting that CD74 expressed in microglia might be pathogenic in AU. CCL5 was reported to directly activate proinflammatory phenotype polarization of macrophages via the activation of NF‐κB pathways. Interestingly, we observed an increase of CCL5 secretion in microglia overexpressing CD74, suggesting that proinflammatory phenotype of microglia elicited by CD74 might be triggered by CCL5.^34^ The blockade of CCL5 effectively attenuated the above alterations in oeCD74 microglial cells, which indicated that microglial activation could be partially modulated by CD74/CCL5/NF‐κB pathway in EAU course.

Cd74 is reported to facilitate the immunochemotactic activity of microglia as a cell‐surface receptor for macrophage migration inhibitory factor (MIF), and Ccl5 as a chemokine, is abundant in inflammatory microglia and can mediate the infiltration of peripheral immunocytes to targeted tissues leading to local inflammation.^27^, ^35^, ^36^ The high enrichment of *Cd74* and *Ccl5* in IAM subgroup at the peak of EAU seemed to be a robust response to the irritation of inflammatory stimuli, and these two specific molecules synergistically promoted the proinflammatory phenotype transition of microglia in a NF‐κB dependent manner to some extent. CD74 might endow microglia with potential immunochemotactic activity resulting in the accumulation of chemokines including CCL5, which could lead to the infiltration of peripheral immune cells into ocular tissues during EAU progression. However, the precise mechanism underlying the synergistic relationship between CD74 and CCL5 in microglial activation and their functions at EAU disease peak still needs further investigation.

At present, drugs (such as Minocycline) and synthetic compounds inhibiting the activation and proliferation of microglia have been applied in the clinical or preclinical trials of a variety of diseases, including Parkinson's disease, schizophrenia, and traumatic brain injury.^37^, ^38^, ^39^ Extensive studies and our research have shown that microglial activation is a key pathological sign of AU closely related to disease severity. We found that CD74 and CCL5 could activate microglia towards proinflammatory phenotype via NF‐κB signaling in vitro, and in vivo experiments further confirmed the potential therapeutic effects of targeting Cd74 and Ccl5, shown as the inhibition of microglial activation and the resolution of EAU disease. Therefore, we provide a potential target and strategy for the treatment of AU via suppressing microglial activation.

In this study, we only obtained CD45‐positive cells, therefore, there may be a certain bias in the capture of immune cell data and a lack of interaction information with critical retinal cell types, including neurons and glial cells. Microglia only account for a small proportion of normal retina tissue, therefore, it is difficult to obtain enough microglia for characterization under healthy conditions. The small size of retina tissues and the strict requirement of scRNA‐seq for cell viability led to the limited number of immune cells obtained as a whole. Increasing the sample size will help to better clarify precise subsets and different functions of microglia under EAU disease conditions.

In conclusion, our evidence indicates the potential pathogenicity of *Cd74* ^high^ *Ccl5* ^high^ microglia with proinflammatory phenotype at disease peak of EAU, and targeting CD74 and CCL5 of activated microglia is a potent therapeutic target for AU.

## 4 MATERIALS AND METHODS

### 4.1 Animals and reagents

Female C57BL/6j mice at 6–8 weeks of age were purchased from Chongqing Medical University. The mice were housed under specific pathogen‐free conditions. Human IRBP_651‐670_ (LAQGAYRTAVDLESLASQLT) was purchased from Shanghai Sangon Biological Engineering Technology & Services Ltd. Co. Pertussis toxin (PTX) and complete Freund's adjuvant (CFA) were obtained from Sigma‐Aldrich. Anti‐CD74 antibody (Abcam, ab245692) and anti‐TMEM119 antibody (Oasis Biofarm, OB‐PGP072) were used in this study. The oeCD74 and vehicle lentivirus were purchased from OBiO Tech. The polyclonal anti‐Cd74 antibody was customized by Suzhou Younuoke Biotechnology Co., Ltd. The neuCCL5 antibody (RD, MAB678), IgG_1_ isotype control (RD, MAB002), neuCcl5 antibody (RD, MAB478), IgG_2A_ isotype control (RD, MAB006) were also obtained. All primers were synthesized by Shanghai Sangon Biological Engineering Technology and Services (Table S4). The EasySep Mouse PE CD45 Positive Selection Kit was purchased from Stem Cell (#18554).

### 4.2 Induction of EAU animal model

Note that, 500 mg human IRBP_651−670_ was dissolved in 1 mL phosphate‐buffered saline (PBS) containing 20% DMSO that was emulsified 1:1 (v/v) in CFA containing 40 mg M. tuberculosis strain H37Ra. Mice were first anesthetized and immunized subcutaneously with 500 µg human IRBP_651−670_. Then 1 µg of PTX was injected intraperitoneally. Eyes were dilated with tropicamide (0.5%) and observed for clinical manifestations using a slit‐lamp microscope at each time point during EAU. The eyes were fixed in 4% buffered paraformaldehyde at 37°C for 12 h. Serial 4–6 µm sections were collected through the pupillary–optic nerve axis and stained with hematoxylin and eosin to evaluate the pathological score. Clinical and histopathological assessments were blindly scored by two independent ophthalmologists based on EAU criteria described previously.^40^, ^41^

### 4.3 Tissue dissociation and cell processing

The retinas of four mice per group at each time point were collected into one sample for subsequent sequencing. Retinal tissues were collected under a dissecting microscope and rinsed with PBS. The retinas were digested into a single‐cell suspension using 0.25% trypsin at 37°C for 30 min and neutralized by Complete Dulbecco's modified Eagle's medium (DMEM). The single‐cell suspensions were filtered with a 40 µm cell strainer. CD45^+^ cells were sorted by magnetic beads. Acridine orange/propidium iodide dye was used for cell counting and ensuring cell viability reaching approximately 90%. Single‐cell suspensions were then subjected to 10×Genomics scRNA‐seq.

### 4.4 scRNA‐seq library construction

The single‐cell suspensions, gel beads, and oils were added to the 10×Genomics single‐cell A chip. The samples were transferred into PCR tubes after droplet generation. Reverse transcription was conducted using a T100 Thermal Cycler (Bio‐Rad), and then cDNA was recovered utilizing a recovery agent. After clean‐up using SPRIselect beads, the cDNA was amplified for 10 cycles. The concentration of cDNA was measured with a Qubit2.0 fluorometer (Invitrogen). Single‐cell cDNA libraries were prepared based on the Chromium Single Cell 3′ Reagent Kit v2 user guide.

### 4.5 Data processing

Single cells were captured using a 10×Genomics with the v2 single cell‐reagent kit. The reads were aligned to the mouse genome using the Cell Ranger toolkit (version 2.1.1) provided by 10×Genomics and then used for a unique molecular identifier (UMI) counting. Preliminary processing and filtration of single cells were performed using Cell Ranger (version 2.1.1).

Subsequently, the filtered data and their UMI count matrices were imported into Seurat (version 2.1.0) for further analysis. Then, we performed a preliminary filtering of cells and genes with two parameters (min.cells = 3, min.features = 200). Additionally, cells with a mitochondrial percentage > 5 were removed. We normalized the data using the Log Normalize method. We used the parameter vars.to.regress in the ScaleData function to regress the mitochondrial percentage out of the scaled data and also used the Seurat function Find Variable Genes to identify variable genes with a low x cutoff of 0.0125, a high x cutoff of 3 and a y cutoff of 0.5.

### 4.6 Canonical correlation analysis

Canonical correlation analysis (CCA) was applied to correct for sample‐specific effects and to mine the correlations between sets of data. Next, we integrated the four samples and removed the batch effect with the Run Multi CCA function in Seurat.

### 4.7 Dimension reduction method and unsupervised clustering analysis

Clustering at a resolution of 0.38 was performed on expression data processed with the cca.aligned package for the top 14 aligned CC dimensions using the graph‐based shared nearest neighbor (SNN) method. First, the k‐nearest neighbors were calculated, and the SNN graph was constructed. Second, the modularity function was optimized to determine the clusters. Third, t‐distributed stochastic neighbor embedding (t‐SNE), a common nonlinear dimension reduction method, was performed in this study. We used the Seurat function Run t‐SNE to achieve dimension reduction.

### 4.8 Identification of cell types

We first identified the DEGs in each cell type and compared each cluster to all others combined using the Wilcoxon method in the Seurat function Find Markers to identify cluster‐specific marker genes. Finally, we manually annotated the information for each cell type based on these classical markers.^21^, ^33^, ^42^

### 4.9 Trajectory analysis

Trajectory analysis (Monocle version 2.2.0) was used to explore the transitions among three microglial subtypes on D0 and D14.^43^ Therein, NIM was used as the initial root state. A CellDataSet object was created using the data from clustering analysis. an unsupervised gene list including the top 2000 variably expressed genes from clustering analysis was selected to construct a single‐cell trajectory. After reducing the dimensionality of the data with DDRTree, the cells were ordered into pseudo‐time. A branch of the whole pseudo‐time trajectory was extracted, and a separate trajectory was constructed on microglia subclusters.

### 4.10 Pathway analysis

GO and KEGG pathway enrichment analyses were performed on target differential genes. These enrichment analyses were performed using the clusterProfiler (version 3.16.0) R package.^44^ The P value was corrected by the Benjamini and Hochberg method.

### 4.11 Electroretinogram recording

ERG was performed in D0 control and D14 EAU mice to evaluate retinal functional changes. Mice were dark‐adapted for at least 12 h and then anesthetized. Prior to the test, the pupils were dilated with 1% tropicamide. Two active gold electrodes were used as recording electrodes, and the reference and ground electrodes were placed on each cornea and subcutaneously inserted into the mid‐frontal areas of the head and tail, respectively. Light stimuli were delivered at 3.0 cd s/m^2^ with a xenon lamp. A RETI‐Port device (Roland Consult) was used for recording and processing the b‐wave amplitudes. All procedures were finished in a dark room with a dim red safety light.

### 4.12 Real‐time quantitative PCR

Retinal tissues were collected from D0 control mice and D14 EAU mice, and total RNA was isolated using an RNA extraction kit (Accurate Biology, AG21023). For RT‐qPCR, total RNA was reverse‐transcribed using RT Master Mix for qPCR (MCE, HY‐K0510). cDNA was quantified using primers specific for mice in an ABI 7500 real‐time PCR system (Applied Biosystems). PCR amplification was performed in a volume of 20 µL using the SYBR Green qPCR Master Mix (MCE, HY‐K0501). The specific transcripts were confirmed by assessment of melting curve profiles at the end of each PCR round. The results were analyzed based on group assignments. β‐Actin was used as the internal control, and the results were calculated using the ^ΔΔ^Ct method.

### 4.13 Immunofluorescence staining

Eyeballs obtained at different time points during EAU were fixed for 2 h in 4% paraformaldehyde. The retinas were extracted and shredded into flat mounts, then placed into 0.4% Triton X‐100 and 5% goat serum for blocking for 1 h, and incubated with primary antibodies at 4°C overnight. After washing, the retinas were incubated with secondary antibodies at 37°C for 1 h. Images were captured by confocal microscopy (Leica). The following primary antibodies were used: anti‐TMEM119 (1:500).

Retina paraffin sections were fluorescently immunolabeled with the following primary antibodies: anti‐CD74 (1:300), anti‐CCL5 (1:300), and anti‐TMEM119 (1:500) and with appropriate secondary antibodies. Images were taken by PANNORAMIC (3DHISTECH) and analyzed by Halo v3.0.311.314 (Indica Labs).

### 4.14 Cell culture and treatment

For HMC3 cells, a complete minimum essential medium containing 10% fetal bovine serum (FBS), 1% penicillin/streptomycin, and 50 µg/mL streptomycin were used for their culture. HMC3 cells were seeded at 3 × 10^5^ cells per well in 6‐well plates for 24 h, and then randomly divided into the Control and LPS (1 µg/mL) + IFN‐γ (0.5 µg/mL) groups for 24 h. Finally, cells and supernatant were collected to detect specific indicators.

For BV2 cells, DMEM/F‐12 supplemented with 10% FBS and 1% penicillin/streptomycin were used for their culture. The cells were seeded at 3 × 10^5^ cells per well in 6‐well plates for 24 h, and then stimulated without or with LPS (1 µg/mL). Another 24 h later, Cells and supernatant were collected for the following measurements.

### 4.15 Cell transfection for overexpression

HMC3 cells were transfected with oeCD74 or vehicle lentivirus (MOI = 30) for 8 h, and cultured for 2 days after changing the medium. After assessing transfection efficiency by fluorescence microscope, cells were cultured with puromycin (2 µg/mL)‐containing medium. The cells were seeded at 3× 10^5^ cells per well in 6‐well plates. 24 h later, cells and supernatant were collected to measure the specific indicator. For studying the function of Ccl5, the cells received neuCCL5 (1 µg/mL) and IgG administration for 24 h. Another 24 h later, cells and supernatant were collected.

### 4.16 Enzyme‐linked immunosorbent assay

The supernatant of HMC3 cells after the corresponding treatment was retained. The levels of IL‐6 and CCL5 in the supernatant were measured using human enzyme‐linked immunosorbent assay kits (R&D Systems).

### 4.17 Western blotting

Radio‐immune precipitation assay lysis buffer (Beyotime, P0013B) was used for tissue and cell lysates. BCA assay kit (Beyotime, P0012S) was used for protein concentration determination. After 30 µg protein amount was separated using 4%–20% sodium dodecyl sulfate‐polyacrylamide gel electrophoresis, it was transferred to polyvinylidene difluoride membranes. The membranes were blocked with 5% skim milk at 37°C for 2 h and then incubated with primary antibodies at 4°C overnight and secondary antibodies at 37°C for 1 h. After washing, the membranes were visualized by an ECL Kit (MedChemExpres) and quantified with ImageJ software. All experiments were repeated in triplicate for statistics. Following primary antibodies were used: anti‐iNOS (1:1000, Proteintech), anti‐COX‐2 (1:1000, Proteintech), anti‐IL‐6 (1:1000, Proteintech), anti‐TLR4 (1:1000, Proteintech), anti‐MYD88 (1:1000, Proteintech), anti‐NF‐κB‐p65 (1:1000, Affinity), anti‐NF‐κB‐p‐p65 (1:1000, Affinity), anti‐CD74 (1:1000, Abcam), β‐Actin (1:2000, Abcam).

### 4.18 Subretinal injections

Subretinal injections were performed on EAU mice on D8. The mice were fully anesthetized first and dilated pupils and an aperture was then made at the limbus with a 30‐gauge needle under a dissecting microscope. Next, a blunt 33‐gauge needle was gently inserted through the aperture without lens damage. Each eye was injected with 0.5 µL of the polyclonal anti‐Cd74 antibody, neuCcl5 antibody, or IgG antibody. Visible subretinal blebs were observed in the injected area to ensure that these antibodies were successfully injected into the retina. All animals received antibiotic ointment on their corneas and were observed daily after the operation.

### 4.19 Statistical analysis

All data are presented as the means ± SDs. Statistical analysis was performed using SPSS 20.0. The numbers in the figure legends represent independent biological replicates. According to their normality, an unpaired Student's t‐test or Mann‐Whitney U test was applied to compare between two groups, and a one‐way analysis of variance or Kruskal‐Wallis test was used for multiple groups (* *p *< 0.05, ** *p* < 0.01).

## AUTHOR CONTRIBUTIONS

Conceiving and study designing: X.L., Y.W., and S.H. Analyzing the data: Z.X., J.L., X.L., N.L., and H.Z. Conducting experiment: J.L., X.L., H.Z., Y.L., Z.Z., and G.W. Writing: J.L., X.L., Y.W., and S.H. All authors have read and approved the final manuscript.

## CONFLICT OF INTEREST STATEMENT

The authors declare no conflict of interest.

## ETHICS STATEMENT

This study was conducted according to the Association for Research in Vision and Ophthalmology (ARVO) Statement. The protocol of this study was approved by the Ethics Committee of the First Affiliated Hospital of Chongqing Medical University (2019‐101). All efforts were made to minimize mouse suffering.

## Supporting information

Supporting Information

Supporting Information

Supporting Information

## Data Availability

All data supporting the findings of this study are available from the authors upon reasonable request. The scRNA‐seq data are openly available in the Gene Expression Omnibus (GSE191260).
