# Supplementary material for: Single‐cell RNA sequencing reveals inflammatory retinal microglia in experimental autoimmune uveitis
Source: MedComm (2020). 2024 Apr 7;5(4):e534. [Article in Italian] doi: 10.1002/mco2.534 (PMC10999176; doi:10.1002/mco2.534)
Supplement: Supplementary file 1 — Supporting Information [file MCO2-5-e534-s002.docx]

**Single-cell RNA Sequencing Reveals Inflammatory Retinal Microglia in Experimental Autoimmune Uveitis**

Jiangyi Liu^1 #^, Xingyun Liao^1,2 #^, Na Li^3^, Zongren Xu^1^, Wang Yang^4^, Hongxiu Zhou^1^, Yusen Liu^1^, Zhi Zhang^1^, Guoqing Wang^1^, Shengping Hou^1,5*^

^1^The First Affiliated Hospital of Chongqing Medical University; Chongqing Key Laboratory for the Prevention and Treatment of Major Blinding Eye Diseases; Chongqing Eye Institute, Chongqing 400016, China.

^2^Department of Medical Oncology, Chongqing University Cancer Hospital, Chongqing 400030, China.

^3^Department of Laboratory Medicine, Beijing Tongren Hospital, Capital Medical University, Beijing 100005,China.

^4^Department of Kidney, First Affiliated Hospital, Third Military Medical University (Army Medical University), Chongqing 400038, China.

^5^Beijing Institute of Ophthalmology, Beijing Tongren Eye Center, Beijing Tongren Hospital, Capital Medical University, Beijing Ophthalmology & Visual Sciences Key Laboratory, Beijing, 100730, China.

*Corresponding author: Shengping Hou Ph.D, The First Affiliated Hospital of Chongqing Medical University, Chongqing 400016, China; Beijing Institute of Ophthalmology, Beijing Tongren Hospital, Capital Medical University, Beijing, 100730, China.

Email address: sphou828@163.com; Telephone number: +86-10-58265906

^#^These authors contributed equally to this work.

**Table S1. Marker genes for annotation**

| **Cell type** | **Marker genes** |
| --- | --- |
| Microglia | P2ry12, Tmem119 |
| T Cell | Cd3d, Cd3g, Cd3e |
| Monocyte/Macrophage | Ccr2, S100a4, Ms4a4c, Msrb1 |
| B Cell | Ly6d, H2-DMb2, Mzb1 |
| Plasma Cell | Sdc1, Cd79a, Cd24a |

**Table S4. The primer sequences of RT-qPCR**

| **Primers (Mouse)** | **Forward** | **Reverse** |
| --- | --- | --- |
| IL-1β | ACAAGGAGAACCAAGCAACGA | GTGGGTGTGCCGTCTTTCAT |
| IL-6 | TGATGGATGCTACCAAACTGGA | TCTCTCTGAAGGACTCTGGCT |
| IL-17 | TCAGACTACCTCAACCGTTCCA | CTTTCCCTCCGCATTGACACA |
| TNF-α | TCAACCTCCTCTCTGCCGT | CTCCAAAGTAGACCTGCCCG |
| IFN-γ | CGGCACAGTCATTGAAAGCCTA | GTTGCTGATGGCCTGATTGTC |
| Cd74 | CGAAATCTGCCAAACCTGT | CCCAAGGAGCATGTTATCCA |
| Ccl5 | CTGTCATTGCTTGCTCTAGTCCTA | AATGCTGATTTCTTGGGTTTGCT |
| β-Actin | GATGACATGGTGAAGACGGC | AGGCACAGGGTCATCATCAA |


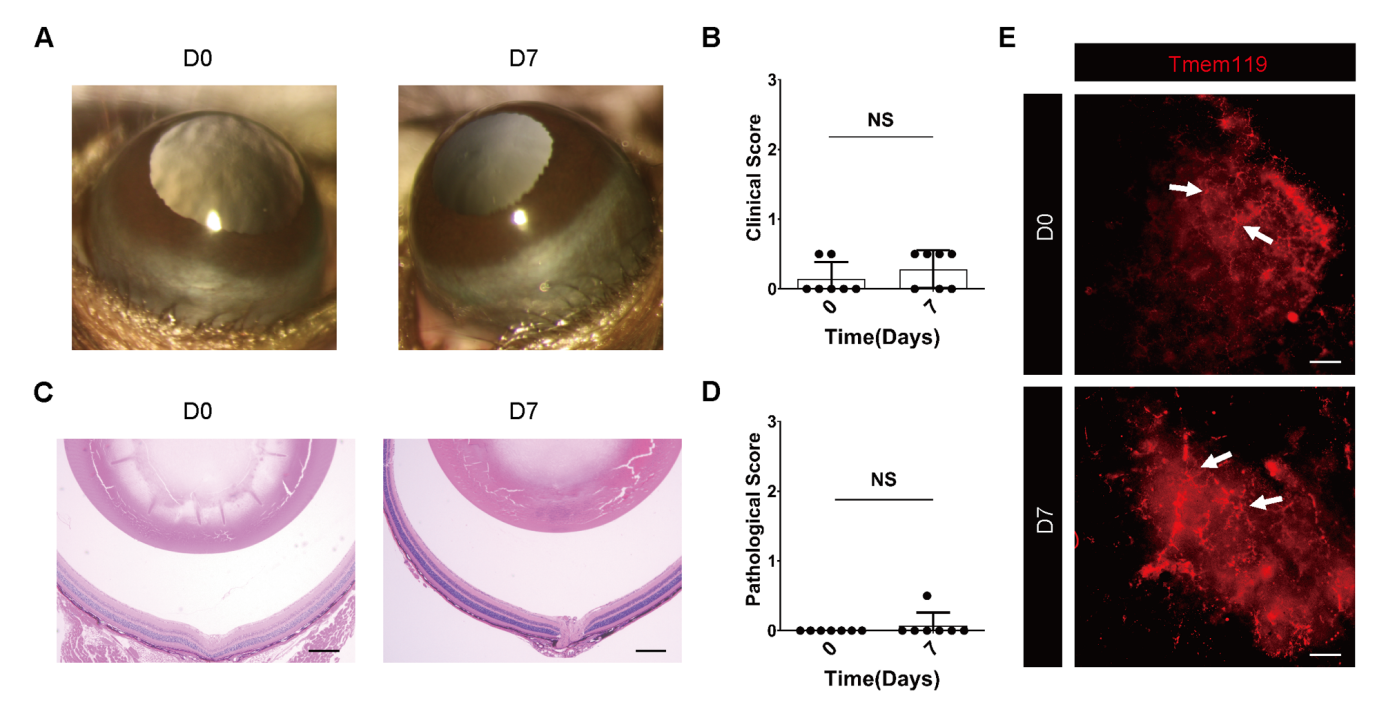


**Figure S1. Identification of EAU phenotype on D7.** (A and B) The clinical [sympto](javascript:;)ms and scores in the D0 group and D7 EAU group. *n* = 7 mice per group. (C and D) Retinal histopathological staining and scores in the D0 group and D7 EAU group. *n* = 7 mice per group. (scale bar, 200 μm). (E) Retinal microglia (Tmem119^+^) staining in the D0 group and D7 EAU group. (scale bar, 20 μm; white arrows, ramified microglia). NS: no significance.


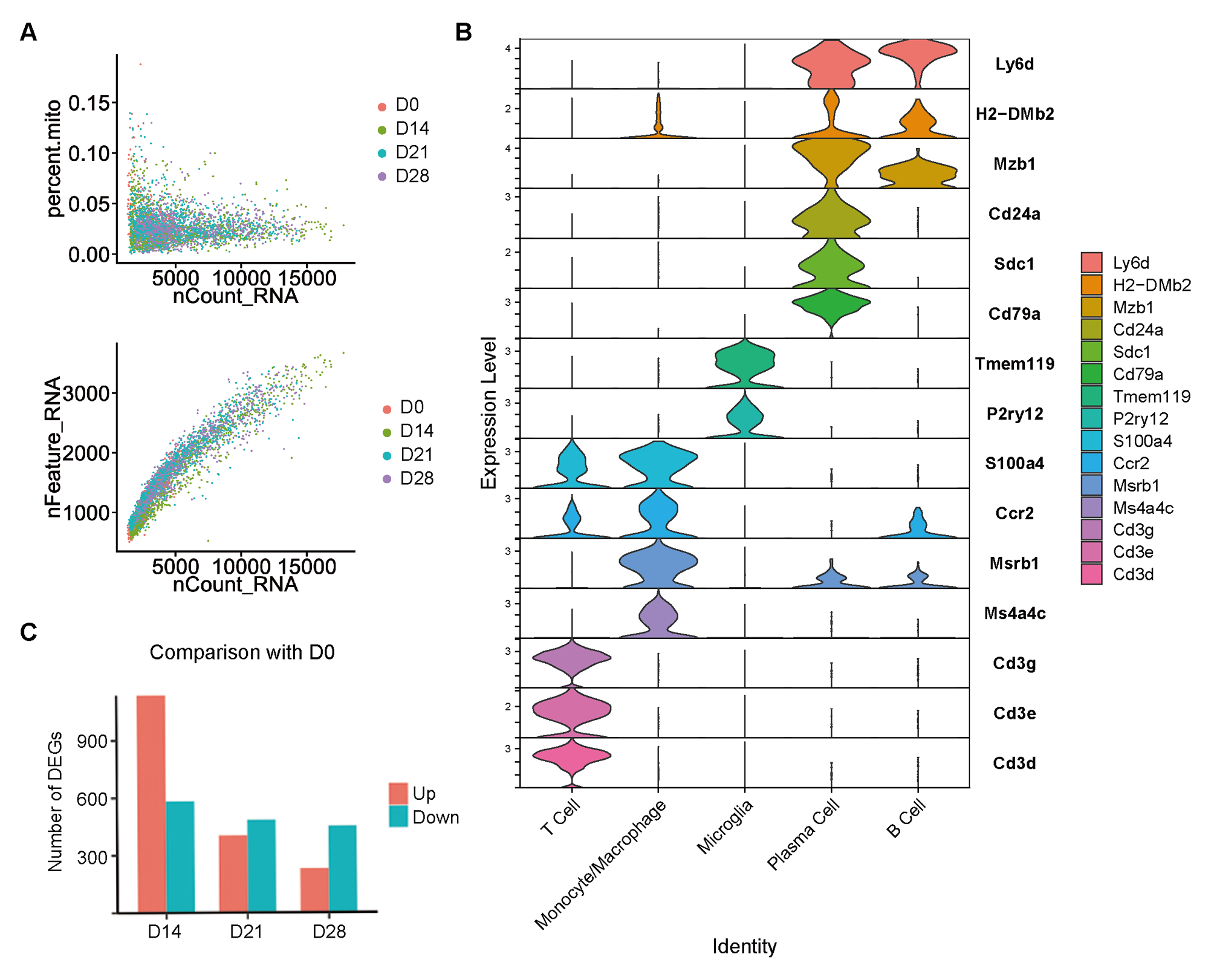


**Figure S2. Related information of retinal immune cells in EAU scRNA-seq data.** (A) Quality control of scRNA-seq data. (B) Vlnplot of canonical lineage marker genes expressed in each cell type during EAU. (C) Histogram of the number of upregulated and downregulated DEGs on D14, D21, and D28 compared with D0. The numbers of altered genes with log fold-change (FC) values >0.5 and adjusted *p* values <0.05 at each time point.


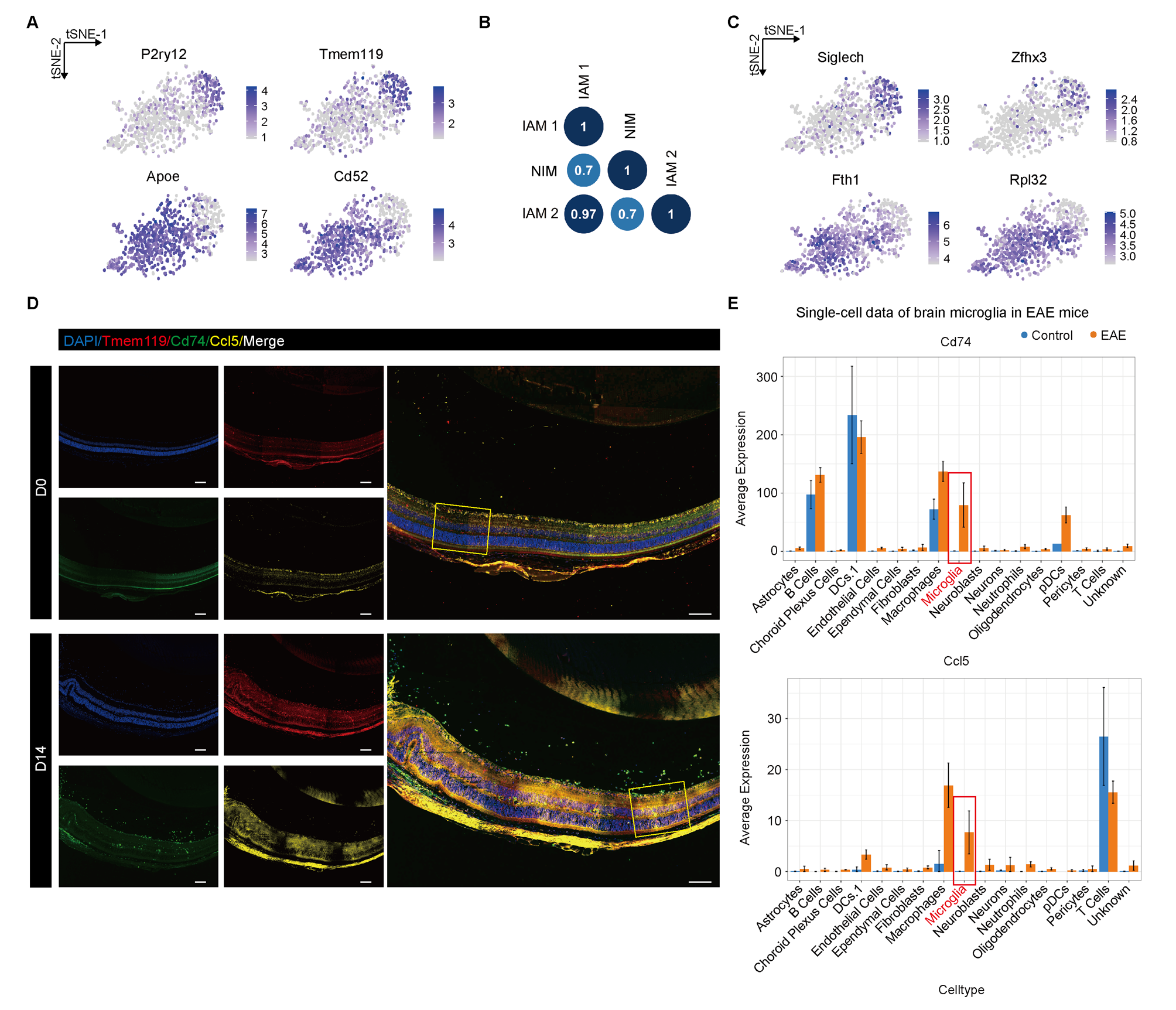


**Figure S3. Inflammation-associated microglia in the EAU retina.** (A) t-SNE plots of homeostatic and activated microglial marker genes expressed in microglial subpopulations on D0 and D14. (B) Plot of the relationship between the microglial subpopulations on D0 and D14. All coefficients were greater than 0.5. (C) t-SNE plots of several genes expressed in microglial subpopulations on D0 and D14. (D) Representative images of retinal microglia (Tmem119^+^) co-stained with Cd74 and Ccl5 in the D0 group and D14 EAU group. (scale bar, 100 μm; yellow box, enlarged images shown in Figure 3F). (E) Histograms of the expression of *Cd74* and *Ccl5* in brain microglia of EAE mice.


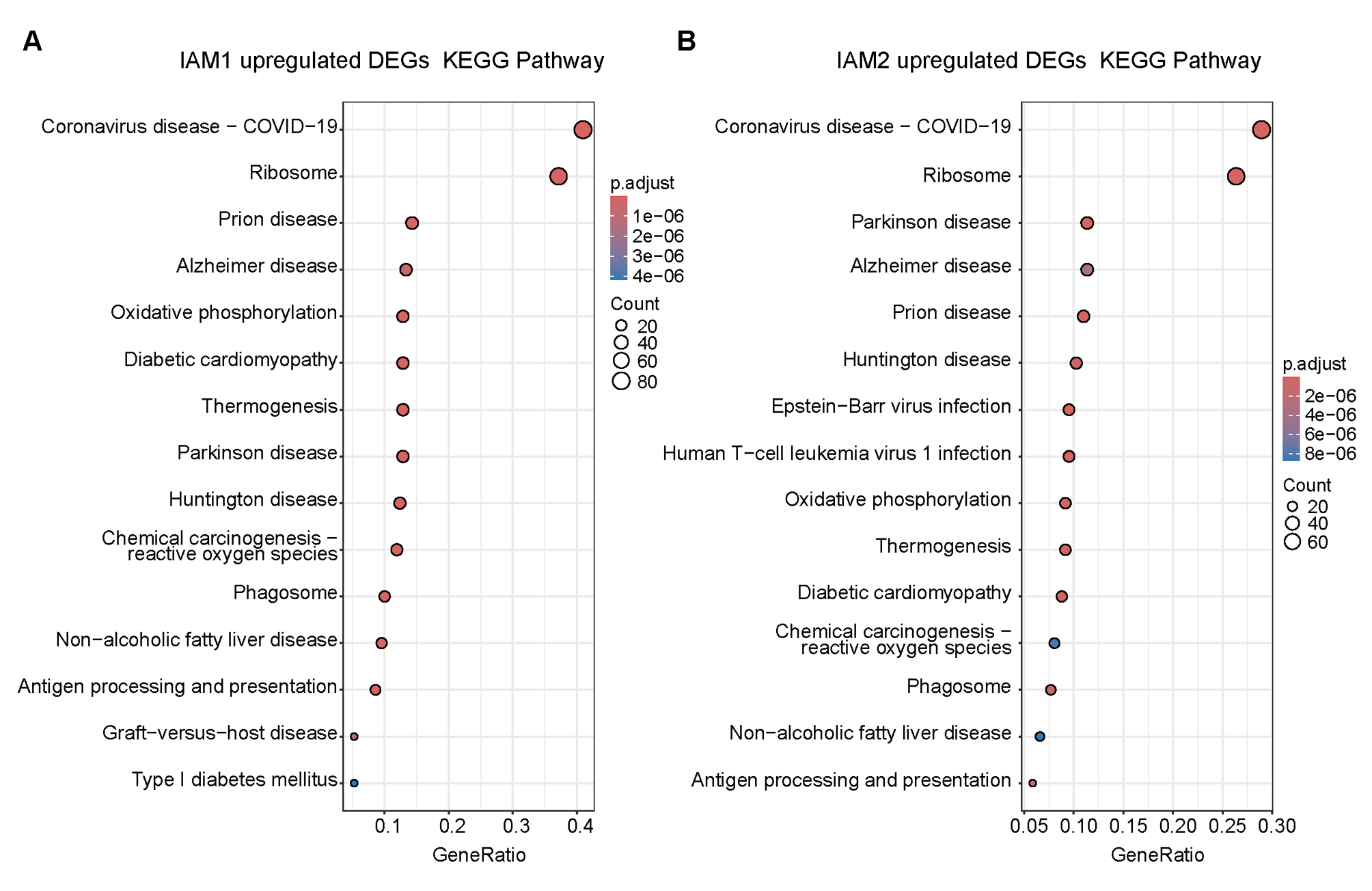


**Figure S4. KEGG analysis of retinal microglia subpopulations in EAU on D0 and D14.** (A and B) Dot plots of the top KEGG pathways calculated by using upregulated DEGs in (A) IAM1 and (B) IAM2 subpopulations compared with NIM.


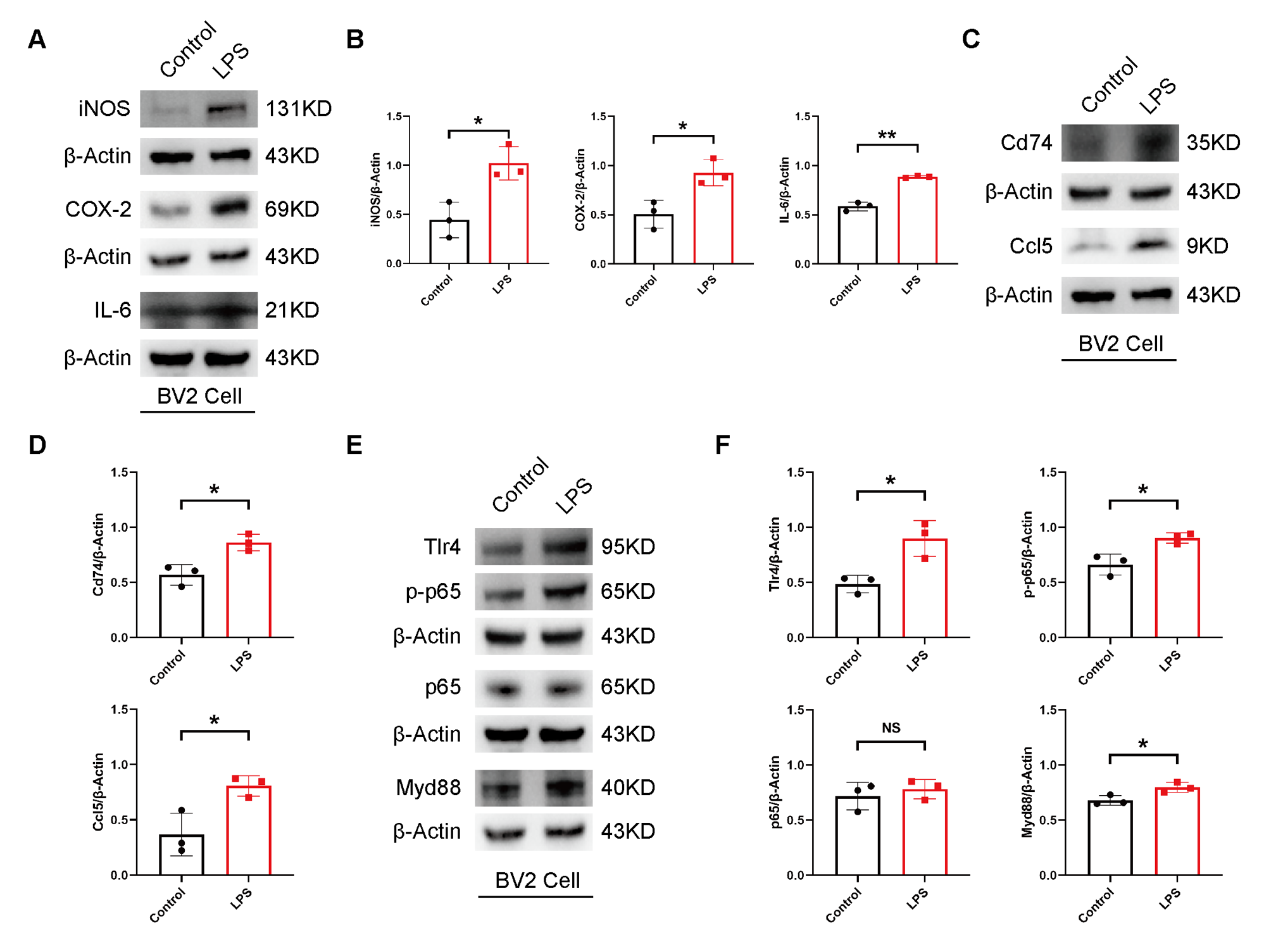


**Figure S5. Microglial changes under inflammation stimulation *in vitro*.** (A and B) The protein expression of proinflammatory microglial markers (IL-6, COX-2, iNOS) in the Control and LPS-induced BV2 cells. (C and D) The protein expression of Cd74 and Ccl5 in the Control and LPS-induced BV2 cells. (E and F) The protein expression of Tlr4 signaling factors in the Control and LPS-induced BV2 cells. * *p* < 0.05, ** *p* < 0.01, NS: no significance.
